# Employing Advanced Technology to Reduce Postoperative Pancreatic Fistula

**DOI:** 10.1097/AS9.0000000000000373

**Published:** 2024-01-09

**Authors:** Brian A. Boone

**Affiliations:** * From the Department of Surgery, West Virginia University, Morgantown, WV; † Department of Microbiology, Immunology and Cell Biology, West Virginia University, Morgantown, WV.

Despite technical improvements over preceding decades, postoperative pancreatic fistula (POPF) continues to be a significant source of morbidity for patients undergoing pancreatectomy, driving postoperative complications,^1^ impacting length of stay,^2^ delaying adjuvant treatment,^3^ and reducing survival.^4^ As a result, strategies to assess the risk of POPF and reduce its occurrence are critically important to improving the care of patients undergoing pancreatic resection.

The recent expansion in the robotic approach to pancreatic surgery has been associated with reduced risk of POPF in select studies,^5–7^ however, the loss of haptic feedback remains a major limitation of the current robotic technology and hinders complex anastomoses such as pancreaticojejunostomy. In this proof of concept study by Chen et al,^8^ the authors utilize near-infrared fluorescence imaging with indocyanine green to assess hypoperfusion of the pancreatic stump during robotic pancreaticojejunostomy. Development of hypoperfusion after tying transpancreatic sutures was associated with a 67% risk of grade B/C POPF versus only 17% in those without hypoperfusion. These findings have important ramifications for robotic pancreatoduodenectomy, but also may be applicable for open surgical technique. Notably, this experience was reported even after the often considered robotic “learning curve” of the operative surgeon, who has expansive experience with minimally invasive pancreatoduodenectomy, confirming a role for continuous technical assessment and improvement even for seasoned expert surgeons.

Importantly, the authors applied their findings to modify their technique, reducing the POPF rate in subsequent patients by using a “measured” tension approach. This work represents another excellent example of how the ease of video review with robotic surgery can drive quality improvement and support technical advancement of robotic pancreatoduodenectomy.^9–13^ Further, this approach may not just drive future improvements, but monitoring pancreatic stump perfusion in real-time in the operating room could support modifying the anastomosis if necessary.

Further studies are needed to expand these findings in a prospective fashion, which will allow for multivariable analysis to determine whether clinical factors including gland texture or duct size may outweigh and/or influence suture tightness. It is possible that soft pancreata may influence the knot tightness and subsequent perfusion more than the technical performance of the operative surgeon. Additionally, these studies would also validate the impact of the transpancreatic sutures on hypoperfusion and leak rate beyond the experience of this single surgical team. Nonetheless, the current work represents an exciting incorporation of emerging technology for technical improvement that is readily available and seamless to utilize during robotic procedures and highlights an important technical factor to consider during robot or open pancreaticojejunostomy.
